# Supplementary material for: Remdesivir Influence on SARS-CoV-2 RNA Viral Load Kinetics in Nasopharyngeal Swab Specimens of COVID-19 Hospitalized Patients: A Real-Life Experience
Source: Microorganisms. 2023 Jan 25;11(2):312. doi: 10.3390/microorganisms11020312 (PMC9959460; doi:10.3390/microorganisms11020312)
Supplement: Supplementary file 1 [file microorganisms-11-00312-s001.zip › microorganisms-1960431-supplementary.pdf]

Supplementary material

Table S1. Comparison of Ct values and Delta Ct in survivors vs nonsurvivors COVID-19 patients. Independent Samples Test statistics, p values and effect size.

**Mann-Whitney test**	**W**		**p**	**Rank-Biserial Correlation**
Ct E T0	5.270.000		**< 0.001**	0.349
Ct N T0	5.268.000		**< 0.001**	0.349
Ct RdRp T0	5.157.000		**0.002**	0.320
Ct E T7	5.742.000		**< 0.001**	0.470
Ct N T7	5.858.500		**< 0.001**	0.500
Ct RdRp T7	5.848.000		**< 0.001**	0.497
ΔCt E	4.609.000		0.084	0.180
ΔCt N	4.704.500		**0.050**	0.204
ΔCt RdRP	4.865.000		**0.018**	0.246
**Student T-Test**	**t**	**df**	**p**	**Cohen's d**
Ct E T0	3.018	251	**0.003**	0.543
Ct N T0	3.502	251	**< 0.001**	0.630
Ct RdRp T0	2.712	251	**0.007**	0.488
Ct E T7	4.884	251	**< 0.001**	0.879
Ct N T7	5.257	251	**< 0.001**	0.946
Ct RdRp T7	5.271	251	**< 0.001**	0.949
ΔCt E	1.810	251	0.072	0.326
ΔCt N	2.274	251	**0.024**	0.409
ΔCt RdRP	2.692	251	**0.008**	0.484

For the Mann-Whitney test, effect size is given by the rank biserial correlation.
Effect size is considered small for Rank-Biserial Correlation values ranging from 0.1 to 0.3; medium from 0.3 to 0.5; large from 0.5 to 1.
For the Student T test, effect size is given by the Cohen's d.
Effect size is considered small if Cohen's d values = 0.2; medium if d = 0.5; large if d = 0.8.
df: degree of freedom.

Table S2. SARS-CoV-2 viral parameters of the study population according to the treatment received, divided by disease severity.

**Non-severe patients**	**All patients (N=150)**	**Remdesivir [R] (N=43)**	**Protease inhibitors [PI] (N=53)**	**No treatment [NT] (N=54)**	**p**
			**T0 NPh swab**		
Median Ct E	26.66 [20.91-32.65]	27.61 [23.09-30.15]	27.84 [22.53-33.90]	23.73 [18.61-34.77]	0.171
Median Ct N	27.02 [21.65-31.88]	26.30 [22.08-29.92]	29.78 [25.04-34.08]	23.42 [18.61-32.86]	**0.007**
Median Ct RdRP	27.74 [21.92-32.44]	28.35 [23.92-31.48]	28.02 [25.20-32.49]	25.69 [18.99-34.82]	0.415
			**T7 NPh swab**		
Median Ct E	33.72 [27.85-45.00]	33.32 [28.46-37.85]	45.00 [29.79-45.00]	32.11 [24.03-45.00]	**0.046**
Median Ct N	33.41 [27.65-37.64]	32.22 [27.56-36.75]	34.98 [32.20-39.03]	31.10 [32.20-39.03]	**0.018**
Median Ct RdRP	33.76 [28.53-45.00]	33.08 [28.95-39.63]	34.98 [30.69-45.00]	33.00 [25.12-45.00]	**0.204**
			**Viral Decay (T7 – T0)**		
Median ΔCt E	6.99 [0.36-12.50]	6.97 [3.26-10.19]	8.55 [0.00-16.89]	6.26 [0.14-10.83]	0.558
Median ΔCt N	5.66 [2.60-10.17]	4.52 [2.59-10.14]	5.41 [2.63-10.43]	6.65 [2.79-10.66]	0.822
Median ΔCt RdRP	6.51 [1.46-11.94]	7.05 [2.34-10.77]	6.37 [1.41-15.31]	6.28 [0.00-10.12]	0.158
**Severe patients**	**All patients (N=103)**	**Remdesivir [R] (N=80)**	**Protease inhibitors [PI] (N=14)**	**No treatment [NT] (N=9)**	**p**
			**T0 NPh swab**		
Median Ct E	26.06 [21.36-30.89]	25.45 [21.49-30.44]	27.48 [20.42-41.26]	27.31 [20.11-33.22]	0.728
Median Ct N	24.35 [21.15-30.40]	23.88 [21.06-29.27]	29.78 [21.56-34.66]	25.66 [22.61-34.01]	0.181
Median Ct RdRP	26.73 [22.00-31.81]	26.07 [22.34-31.54]	28.30 [20.79-35.58]	27.74 [21.64-34.20]	0.813
			**T7 NPh swab**		
Median Ct E	33.79 [27.25-37.93]	34.00 [29.29-37.18]	28.70 [24.14-45.00]	30.68 [23.68-45.00]	0.753
Median Ct N	31.84 [26.61-35.38]	31.88 [26.92-34.76]	30.01 [26.43-42.78]	30.20 [25.76-39.64]	0.973
Median Ct RdRP	34.28 [28.49-45.00]	34.85 [30.02-38.28]	28.36 [25.02-45.00]	31.10 [24.93-45.00]	0.602
			**Viral Decay (T7 – T0)**		
Median ΔCt E	7.29 [2.18-10.68]	7.94 [3.87-10.65]	5.76 [-1.15-14.40]	3.57 [0.00-6.28]	0.121
Median ΔCt N	6.70 [2.99-10.58]	7.13 [3.12-11.00]	5.90 [0.10-8.02]	3.79 [2.79-7.60]	0.220
Median ΔCt RdRP	7.22 [3.16-10.57]	7.96 [4.58-10.50]	6.68 [-1.14-13.52]	5.39 [0.00-6.37]	0.158

Quantitative data are presented as median [IQR].
Ct: cycle threshold; E: Envelope gene; IQR: interquartile range; N: Nucleocapsid gene; NPh swab: nasopharyngeal swab; RdRP: RNA-dependent RNA Polymerase gene.

Table S3. General characteristics of the study population, restricted to patients who received antiviral treatment within 10 days from symptoms' onset and non-treated patients, overall and divided by treatment groups (remdesivir, protease inhibitors and no antiviral treatment).

	Overall population (N=194)	Remdesivir [R] (N=80; 41.2%)	Protease inhibitors [PI] (N=51; 26.3%)	No treatment [NT] (N=63; 32.5%)	p
**Age: median [IQR]**	63 [51-77]	67 [56.7-76]	67 [51-84]	55 [44.5-75.5]	**0.012**
**Sex: M/F**	119/75 (61/39)	53/27 (66.3/33.7)	27/24 (53.8/46.2)	38/25 (60.3/39.7)	0.357
**Time from symptoms' onset to T0 NPhS: median [IQR]***	5 [2.0-7.0]	5 [3.0-7.0]	4 [1.0-7.0]	6 [1.5-9.5]	0.271
**Non-severe/severe**	125/69 (64.4/35.6)	32/48 (40/60)	39/12 (76.5/23.5)	54/9 (85.7/14.3)	**<0.001**
**ICU admission**	15 (7.7)	11 (13.7)	4 (7.8)	0	**0.009**
**Survivors/non-survivors**	165/29 (85.1/14.9)	65/15 (81.2/18.8)	43/8 (84.3/15.7)	57/6 (90.5/9.5)	0.303
**Corticosteroid treatment**	105 (54.1)	77 (96.2)	13 (25.5)	15 (23.8)	**<0.001**
**Comorbidities**					
Any	167 (86.1)	72 (90)	42 (82.4)	53 (84.1)	0.403
Obesity	30 (15.5)	20 (25)	4 (7.8)	6 (9.5)	**0.009**
Cardiovascular	110 (56.7)	54 (67.5)	28 (54.9)	28 (44.4)	**0.021**
Diabetes	45 (23.2)	23 (28.7)	10 (19.6)	12 (19.1)	0.307
Endocrinologic	24 (12.4)	12 (15)	5 (9.8)	7 (11.1)	0.634
Cerebrovascular	17 (8.8)	11 (13.7)	3 (5.9)	3 (4.8)	0.118
Chronic viral hepatitis	3 (1.5)	1 (1.2)	1 (1.9)	1 (1.6)	0.949
Pulmonary	25 (12.9)	14 (17.5)	7 (13.7)	4 (6.3)	0.139
Renal	17 (8.8)	3 (3.7)	4 (7.8)	10 (15.9)	**0.038**
Solid Tumor	26 (13.4)	14 (17.5)	7 (13.7)	5 (7.9)	0.249
Hematologic	16 (8.2)	9 (11.2)	4 (7.8)	3 (4.8)	0.372
Neurologic/Psychiatric	37 (19.1)	9 (11.2)	13 (25.5)	15 (23.8)	0.066
Rheumatologic	14 (7.2)	5 (6.2)	3 (5.9)	6 (9.5)	0.688
Other	41 (21.1)	11 (13.7)	15 (29.4)	15 (23.8)	0.083

Quantitative data are presented as median [IQR]; qualitative data are presented as absolute frequency (percentage).
*Data for 182 patients, 12 asymptomatic patients were excluded from this analysis.
ICU: Intensive Care Unit; IQR: interquartile range; NPhS: nasopharyngeal swab.

Table S4. SARS-CoV-2 viral parameters of the study population, restricted to patients who received antiviral treatment within 10 days from symptoms' onset and non-treated patients, divided by treatment groups (remdesivir, protease inhibitors and no antiviral treatment).

	**All patients (N=194)**	**Remdesivir [R] (N=80; 41.2%)**	**Protease inhibitors [PI] (N=51; 26.3%)**	**No treatment [NT] (N=63; 32.5%)**	**p**
			**T0 NPh swab**		
Median Ct E	25.90 [20.78-31.20]	26,16 [22.10-30.68]	26.35 [21.82-30.59]	24.40 [18.62-34.55]	0.674
Median Ct N	25.54 [20.56-30.71]	24.58 [21.31-30.47]	28.58 [23.28-31.93]	23.49 [18.70-33.46]	0.060
Median Ct RdRP	26.92 [21.67-32.28]	27.33 [22.60-31.89]	27.56 [22.48-30.78]	25.88 [18.99-34.77]	0.842
			**T7 NPh swab**		
Median Ct E	33.36 [26.38-45.00]	33.82 [29.23-37.94]	34.94 [24.84-45.00]	31.47 [23.85-45.00]	0.409
Median Ct N	32.23 [26.39-37.11]	31.97 [26.92-35.74]	34.60 [27.11-38.37]	30.72 [25.16-37.60]	0.240
Median Ct RdRP	33.40 [26.86-45.00]	34.49 [29.79-45.00]	32.80 [26.20-45.00]	32.88 [25.01-45.00]	0.550
			**Viral decay (T7 – T0)**		
Median ΔCt E	6.97 [1.27-11.71]	7.24 [3.82-10.61]	7.53 [0.00-17.81]	5.72 [0.00-10.53]	0.367
Median ΔCt N	6.30 [2.80-10.37]	6.43 [2.88-10.55]	5.92 [1.84-10.50]	6.60 [2.78-9.89]	0.913
Median ΔCt RdRP	6.61 [2.10-10.67]	7.26 [3.41-10.23]	6.14 [0.65-14.39]	5.91 [0.00-9.37]	0.312

Data reported as median [IQR].

Ct: cycle threshold; E: Envelope gene; IQR: interquartile range; N: Nucleocapsid gene; NPh swab: nasopharyngeal swab; RdRP: RNA-dependent RNA Polymerase gene.

Table S5. SARS-CoV-2 viral parameters of the study population, restricted to patients who received antiviral treatment within 10 days from symptoms' onset and non-treated patients, divided by disease severity.

Non-severe patients	All patients (N=125)	Remdesivir [R] (N=32)	Protease inhibitors [PI] (N=39)	No treatment [NT] (N=54)	p
			**T0 NPh swab**		
Median Ct E	25.68 [19.52-31.15]	27.63 [22.13-30.36]	26.12 [22.09-29.42]	23.74 [18.62-34.77]	0.571
Median Ct N	26.17 [20.75-31.38]	25.77 [21.84-30.65]	28.12 [24.04-31.53]	23.43 [18.61-32.86]	0.158
Median Ct RdRP	26.87 [21.16-31.86]	28.33 [23.00-31.89]	27.14 [22.65-30.38]	25.69 [18.99-34.82]	0.658
			**T7 NPh swab**		
Median Ct E	33.40 [26.43-45.00]	33.52 [30.86-39.68]	45.00 [28.01-45.00]	32.12 [24.03-45.00]	0.247
Median Ct N	33.29 [27.01-37.39]	32.23 [28.77-36.73]	34.98 [30.48-38.37]	31.10 [25.03-37.31]	0.158
Median Ct RdRP	33.12 [27.39-45.00]	33.52 [30.37-41.16]	33.14 [29.46-45.00]	33.00 [25.13-45.00]	0.628
			**Viral decay (T7 – T0)**		
Median ΔCt E	7.02 [2.13-12.95]	7.00 [4.01-11.74]	8.55 [1.45-18.59]	6.26 [0.00-10.84]	0.333
Median ΔCt N	6.04 [2.83-10.90]	5.51 [3.14-10.17]	6.04 [2.74-12.68]	6.66 [2.79-10.67]	0.952
Median ΔCt RdRP	6.37 [1.76-11.70]	7.18 [3.41-11.02]	6.14 [3.05-14.89]	6.29 [0.00-10.13]	0.575
**Severe patients**	**All patients (N=69)**	**Remdesivir [R] (N=48)**	**Protease inhibitors [PI] (N=12)**	**No treatment [NT] (N=9)**	**p**
			**T0 NPh swab**		
Median Ct E	26.07 [21.02-31.53]	25.88 [21.36-30.77]	27.48 [17.93-45.00]	27.31 [20.11-33.22]	0.858
Median Ct N	24.31 [20.51-30.50]	23.83 [20.47-30.44]	29.79 [19.63-34.94]	25.66 [22.61-34.01]	0.380
Median Ct RdRP	26.97 [21.88-32.46]	26.42 [22.33-31.95]	29.59 [18.82-37.25]	27.74 [21.64-34.20]	0.883
			**T7 NPh swab**		
Median Ct E	33.19 [25.90-38.28]	34.03 [29.22-37.79]	24.84 [22.99-45.00]	30.68 [23.68-45.00]	0.625
Median Ct N	31.24 [26.31-36.01]	31.84 [26.54-25.06]	27.72 [25.51-38.36]	30.20 [25.76-39.64]	0.976
Median Ct RdRP	34.28 [26.62-45.00]	34.93 [29.79-45.00]	26.20 [24.14-45.00]	31.10 [24.93-45.00]	0.499
			**Viral decay (T7 – T0)**		
Median ΔCt E	6.76 [0.00-10.48]	7.38 [2.42-10.51]	2.78 [-3.04-13.37]	3.57 [0.00-6.28]	0.173
Median ΔCt N	6.46 [2.46-10.19]	6.56 [2.78-10.92]	5.91 [-2.75-7.85]	3.79 [2.79-7.60]	0.354
Median ΔCt RdRP	6.89 [2.44-10.09]	7.44 [3.72-10.11]	6.68 [-3.17-12.02]	5.39 [0.00-6.37]	0.190

Data reported as median [IQR].
Ct: cycle threshold; E: Envelope gene; IQR: interquartile range; N: Nucleocapsid gene; NPh swab: nasopharyngeal swab; RdRP: RNA-dependent RNA Polymerase gene.
